# LncRNA MCF2L-AS1 promotes malignant progression of colorectal cancer by post-transcriptional activation of MCF2L

**DOI:** 10.1007/s12672-026-04394-6

**Published:** 2026-01-08

**Authors:** Hengchuan Shi, Liang Qiu, Pei Tan

**Affiliations:** 1 Department of Laboratory Medicine, Jiangsu Province official Hospital, No. 65, Jiangsu Road, Nanjing, 210009 Jiangsu China; 2 Jiangsu Health Vocational College, Nanjing, 211800 Jiangsu China

**Keywords:** LncRNA MCF2L-AS1, Heterogenous nuclear ribonucleoprotein D0, Colorectal cancer, Proliferation, Metastasis

## Abstract

**Purpose:**

To elucidate the functional role of MCF2L antisense RNA1 (MCF2L-AS1) in colorectal cancer (CRC) progression and its potential molecular mechanism.

**Methods:**

The expression of MCF2L-AS1/MCF2L in CRC cell lines was detected by qRT-PCR. The biological function of MCF2L-AS1 was investigated in vitro and in vivo (colony formation, CCK8, Apoptosis assays, wound healing and transwell assays, and mouse xenograft models). Mechanistic investigations involved bioinformatics analysis followed by experimental validation using Fluorescence in situ hybridization, RNA pull-down, and RNA immunoprecipitation.

**Results:**

MCF2L-AS1 was notably upregulated in CRC tissues and cells. Functionally, MCF2L-AS1 suppression markedly attenuated the viability and colony capacity of CRC cells, and the number of migratory and invasive cells was markedly reduced both in vivo and in vitro. Mechanism studies have shown that MCF2L-AS1 binds to the heterogeneous nuclear ribonucleoprotein AUF1, facilitating MCF2L protein synthesis through a post-transcriptional regulatory mechanism independent of mRNA stability.

**Conclusions:**

MCF2L-AS1 promotes CRC progression through AUF1-dependent translational regulation of MCF2L expression. MCF2L-AS1 may represents a potential therapeutic target for clinical intervention in CRC.

**Supplementary Information:**

The online version contains supplementary material available at 10.1007/s12672-026-04394-6.

## Introduction

Colorectal cancer (CRC), as one of the commonest primary intestinal malignant tumors, is characterized by high incidence and mortality [1]. Despite continuous improvements in diagnostic techniques and clinical treatment of CRC in recent years, the reality that CRC patients have a poor prognosis has not changed [2]. Hence, genetic and epigenetic alterations and their underlying mechanisms in CRC need to be further explored, to provide effective diagnostic for CRC.

Long non-coding RNAs (lncRNAs) are typically defined as non-coding RNAs with a length exceeding 200 nucleotides [3–5]. Over the past two decades, lncRNAs have been proven to participate in complex biological processes through multiple mechanisms. Among them, are histone modifications, gene expression regulation, chromatin remodeling, interaction with RNA and DNA, and sponging of microRNAs (miRNAs) [6–9]. Extensive research has demonstrated the significant involvement of lncRNAs in the pathogenesis of multiple pathological conditions, particularly in CRC [10, 11].

One important mechanism by which lncRNAs regulate gene expression is by binding to microRNAs (miRNAs) as competing endogenous RNAs (ceRNAs). For example, Xu et al. reported that SNHG1 acts as a sponge for miR-154-5p to regulate the protein D2 (CCND2) and thus regulate the cell cycle [12]. LncRNA UICLM has been proven to promote liver metastasis in CRC by regulating ZEB2 expression as a ceRNA as miRNA-125 [13]. MCF2L-AS1, as a newly discovered lncRNA, has been proven to act as an oncogene and promote the occurrence and development of CRC [14, 15]. However, the molecular mechanisms in current studies are all related to the ceRNA pattern, so we aimed to explore more potential molecular mechanisms of MCF2L-AS1.

Our investigation confirmed that MCF2L-AS1 was significantly upregulated in CRC tissues and cells. More importantly, the data demonstrated that suppression of MCF2L-AS1 effectively restrained proliferative capacity, metastatic potential, and migratory activity of CRC cells in vivo and in vitro. At a mechanistic level, MCF2L-AS1 was located in the cytoplasm and, through direct interaction with AUF1, promotes post-transcriptional activation of MCF2L mRNA, thereby increasing the expression level of MCF2L protein. Overall, this study not only elucidates the functional significance and regulatory networks of MCF2L-AS1 in CRC but also provides valuable insights for developing novel diagnostic biomarkers and therapeutic strategies for CRC.

## Materials and methods

### Cell culture

The human colon epithelial cell line NCM460 and the human CRC cell lines (HCT116, HCT29, HCT8, SW480, SW620, DLD1, and CACO2) were obtained from American Tissue Culture Collection (Manassas, VA, USA). Cellular propagation was performed using Dulbecco’s Modified Eagle Medium (DMEM, Gibco) enriched with fetal bovine serum (10%, Gibco) and antibiotic solution (1% penicillin-streptomycin). Cell cultures were sustained under standardized conditions at 37 °C with 5% CO2.

### Vector construction and cell transfection

MCF2L small interfering RNA (si-MCF2L), AUF1 small interfering RNA (si-AUF1), MCF2L small interfering RNA (si-MCF2L), and negative control (si-NC) were designed and synthesized by RiboBio. The cells were first grown to a density of about 40% in complete culture medium and then transfected with Lipofectamine 3000 (Invitrogen, USA) reagent. Stable expression cell lines were screened first and subsequent experiments were conducted. In addition, the small hairpin RNA (shRNA) of MCF2L-AS1 and its negative controls were purchased from Genechem.

### Western blotting

Cell proteins are collected by RIPA lysis buffer after cleaning with PBS. RIPA lysis buffer is prepared from the ratio of 1:5:5 protein inhibitors, phosphatase inhibitors (KeyGEN), and PMSF. After determining the protein concentration, it was separated on 10% SDS-PAGE gel, and transferred to polyvinylidene fluoride membranes. The skim milk powder and 1 × TBST solution were prepared into 5% skim milk sealer at 1:20 ratio. The membranes were enclosed in skim milk and incubated with diluted specific antibodies overnight, and then incubated with a second antibody. Finally, the protein expression was observed by bioimaging system ECL Plus (Millipore). The detailed information of antibodies was as follows:

β-actin (1: 5000, ab8226, abcam), MCF2L (1:1000, O15068, Affinity), AUF1(1:1000,12770-1-AP, Proteintech), goat antimouse IgG, peroxidase conjugated, H + L (Biosharp, 1:5,000), and goat antirabbit IgG, peroxidase conjugated, H + L (Biosharp, 1:5,000).

### Colony formation assay

HCT116 and HCT8 cellular suspensions were plated in 6-well culture dishes at densities ranging from 500 to 1000 cells per well. Then replace medium every three days until two weeks later. After extracting medium in 6-well plates, the cell colonies were fixed with 4% paraformaldehyde. Subsequently, the fixed colonies were subjected to staining with 5% crystal violet dye solution (Beyotime, China) for visualization. Cell proliferation capacity was assessed by the number of colonies formed.

### CCK-8 assay

Cells were seeded in 96-well plates after being suspended in mediums. The cells were incubated at 37℃, and then 10ul of CCK8 solution was added at five times points, 24 h, 48 h, 72 h,96 h, and 120 h, respectively, and the optical density (OD) of each group at 450 nm was measured to evaluate cell proliferation.

### Wound healing assay

Implanted HCT116 and HCT8 cells into 6-well plates, with a density of 1 × 10^6^ cells per well. Then, use a 200ul sterile gun tip to scratch the cell layer to form a wound, and rinse off non-adherent cells with PBS. The wound area was then photographed under an Olympus FSX100 microscope (Olympus, Japan) at 0 h and 24 h respectively. ImageJ software (Bethesda, USA) was used to measure the area of cell migration between dotted lines to assess cell migration ability.

### Transwell invasion assays

Briefly, HCT116 and HCT8 cells following transfection were seeded into the apical compartment of transwell inserts pre-coated with Matrigel matrix (BD Biosciences, USA). The basal chamber was filled with nutrient-rich medium supplemented with 10–20%FBS. After culture for 24–48 h, the uninvaded cells remaining on the membrane’s upper surface were removed, and then the invaded cells were fixed and stained, and were counted under Olympus FSX100 microscope (Olympus, Japan).

### RNA fluorescence in situ hybridization (FISH)

FISH assay was performed using Flurescent In SituHybridization Kit (Genechem, China) according to the manufacturer’s instructions. Briefly, cells were first washed, fixed, and subjected to a series of treatments before being soaked in a pre-hybridization solution for incubation, followed by incubation with Cy3-labelled probes. Finally, the cell nucleus was stained with DAPI and photographed under a Zeiss microscope.

### Subcellular fractionation

Subcellular fractionation experiment was used PARIS Kit (Invitrogen, USA) to isolate the nuclear and cytoplasmic parts and detect the subcellular localization of RNA.

### Statistical analysis

All statistical analyses were carried out using SPSS 19.0 software (IBM). Differences between the groups were assessed by applying Student’s paired or unpaired *t*-test or one-way analysis of variance (ANOVA), respectively. *P* < 0.05 indicates a statistically significant difference.

A complete description of the methods, including RNA extraction and qRT-PCR, RNA immunoprecipitation assay, RNA pull-down assay, In vivo experiments, Apoptosis assays are available in Supplementary Material.

## Results

### MCF2L-AS1 expression was upregulated in colorectal cancer

**Fig. 1:**
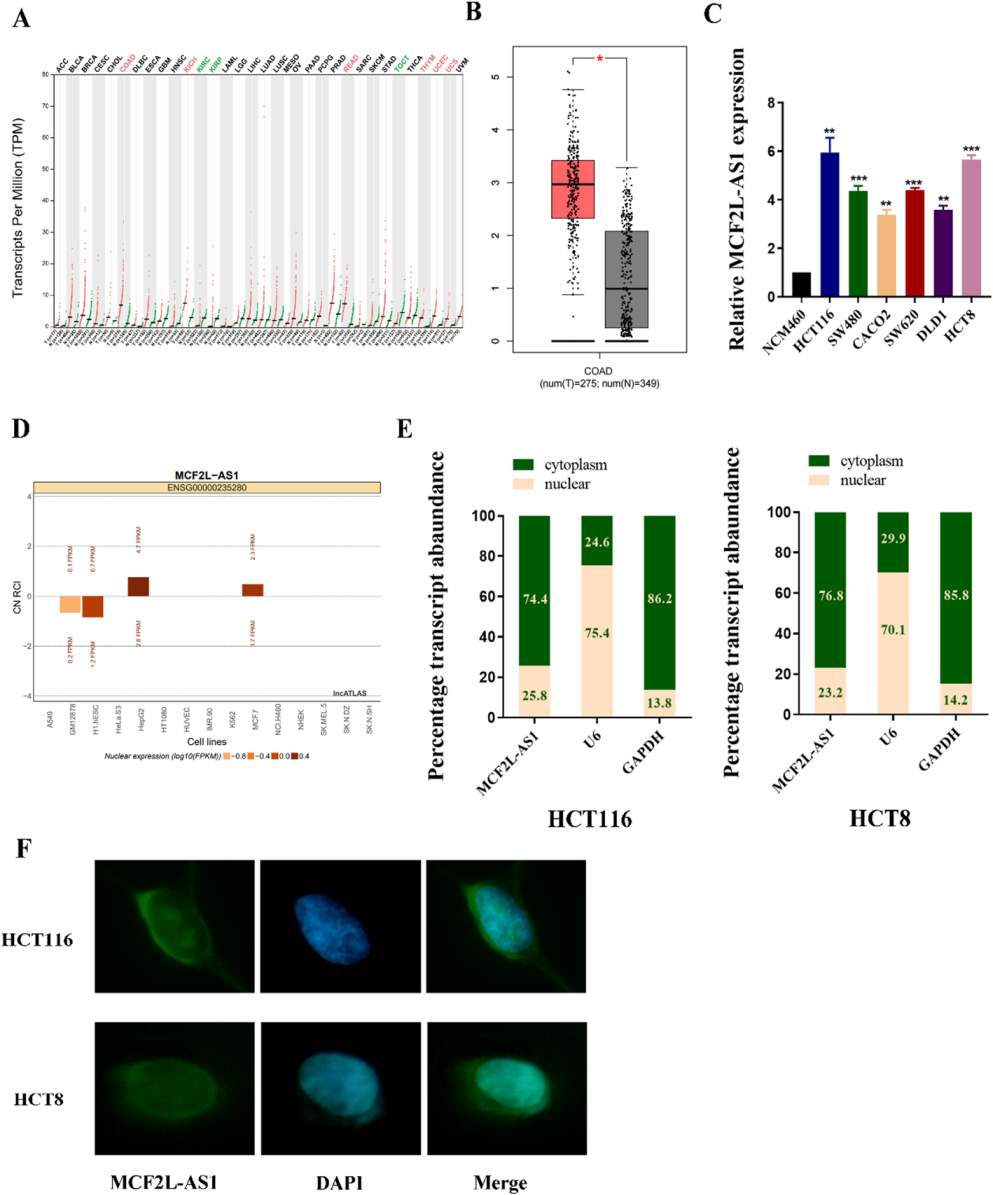
MCF2L-AS1 expression was upregulated in colorectal cancer. **A** The MCF2L-AS1 expression in various tumors. The x-axis represented the sample type and the y-axis represented the MCF2L-AS1 expression. **B** GEPIA of CRC data in TCGA showed MCF2L-AS1 expression state in COAD and normal tissue. **C** qRT-PCR for the expression of MCF2L-AS1 in CRC cell lines. **D** Subcellular localization of MCF2L-AS1 analyzed using the bioinformatics tools in lncATLAS. **E** Relative MCF2L-AS1 expression levels in nuclear and cytosolic fractions of HCT116 cells. Nuclear controls: U6, cytosolic controls: GAPDH. **F** FISH assay identifying the subcellular location of MCF2L-AS1 in the HCT116 cells. *p< 0.05,**p< 0.01, and ***p< 0.001

First, the GEPIA database to examine MCF2L-AS1 expression patterns across multiple tumor types. As illustrated in Fig. 1A–B, MCF2L-AS1 was up-regulated in various tumors, with particularly pronounced elevation observed in CRC tissues compared to corresponding normal tissue. Additionally, qRT-PCR assay was performed in CRC cell lines (HCT116, SW620, SW480, CACO2, DLD1, HCT8), yielding consistent results with the previous findings (Fig. 1C). Subcellular localization analysis utilizing the lncATLAS bioinformatics tool revealed predominant cytoplasmic distribution of MCF2L-AS1 (Fig. 1D). Furthermore, subcellular localization of MCF2L-AS1 in CRC was investigated using FISH and subcellular fractionation assays which revealed its predominant cytoplasmic localization (Fig. 1E–F), consistent with previous results.

### MCF2L-AS1 promotes colorectal cancer cell growth in vitro

**Fig. 2:**
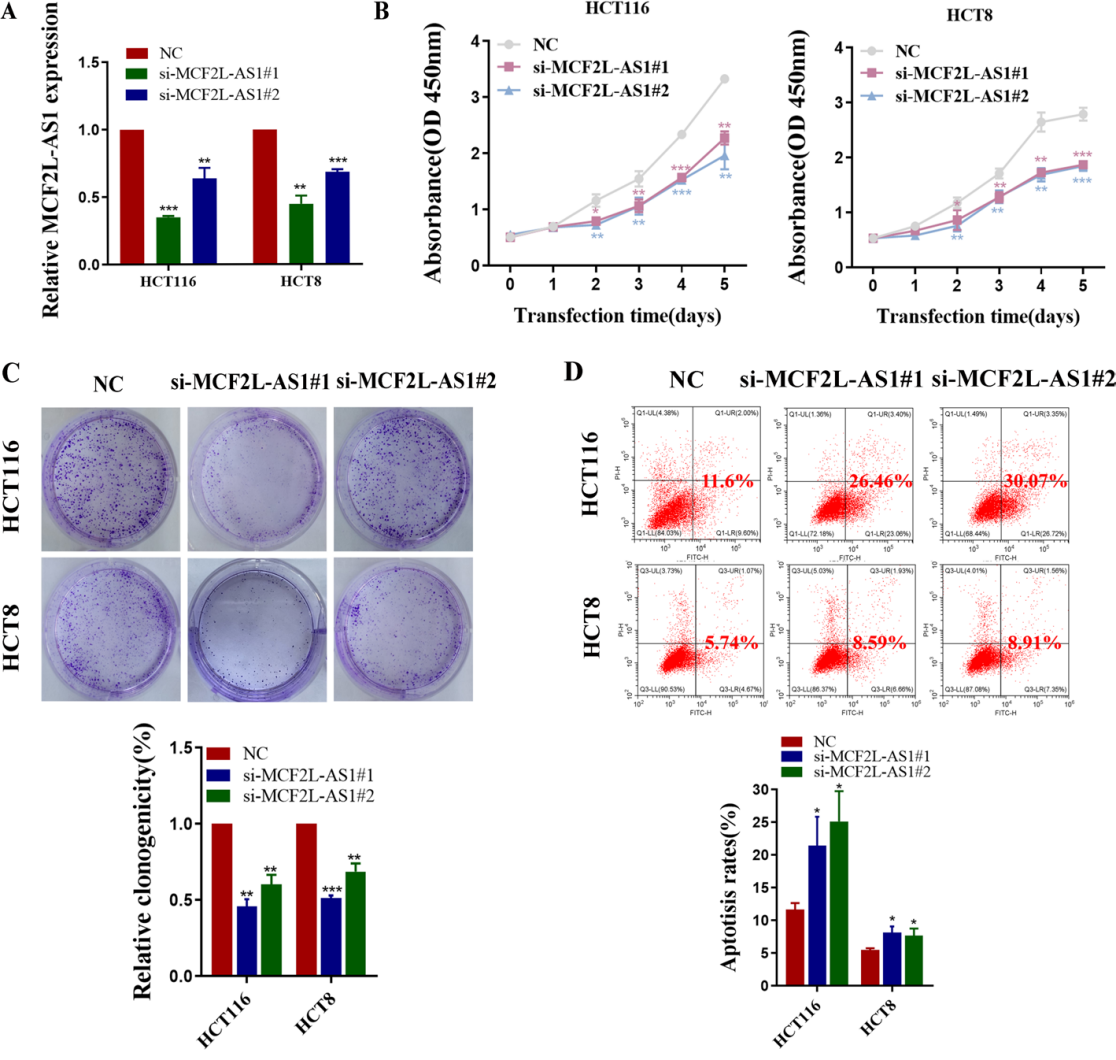
MCF2L-AS1 promotes colorectal cancer cell growth in vitro. **A** The interference efficiency of MCF2L-AS1 in HCT116 and HCT8 cells was tested by qRT-qPCR. **B** CCK8 assays were used to determine the cell proliferation ability of infected cells. **C** HCT116 and HCT8 cells infected with si-MCF2L-AS1 were seeded into six-well plates. The number of colonies was counted on the 14th day after seeding. **D** The effect of MCF2L-AS1 knockdown on cell apoptosis was analyzed by flow cytometry. *p < 0.05, **p < 0.01, and ***p < 0.001

To systematically evaluate the effect of MCF2L-AS1 on CRC cell growth, we conducted a series of in vitro assays. We knock down MCF2L-AS1 to explore its molecular mechanism and the si-MCF2L-AS1 vector was transfected into HCT116 and HCT8 cell lines with high transfection efficiency confirmed by qRT-PCR (Fig. 2A). By performing colony formation and CCK8 assays, the data showed that the downregulation of MCF2L-AS1 diminished the growth viability of HCT116 and HCT8 cells (Fig. 2B-C). We then performed flow cytometry assays to analyze the apoptosis rate. Comparative analysis demonstrated a substantial elevation in programmed cell death rates following MCF2L-AS1 silencing through siRNA transfection, relative to the NC group (Fig. 2D). Collectively, these experimental observations provide evidence that MCF2L-AS1 functionally promotes CRC cell growth in vitro.

### MCF2L-AS1 promotes colorectal cancer cell migration and invasion in vitro

**Fig. 3:**
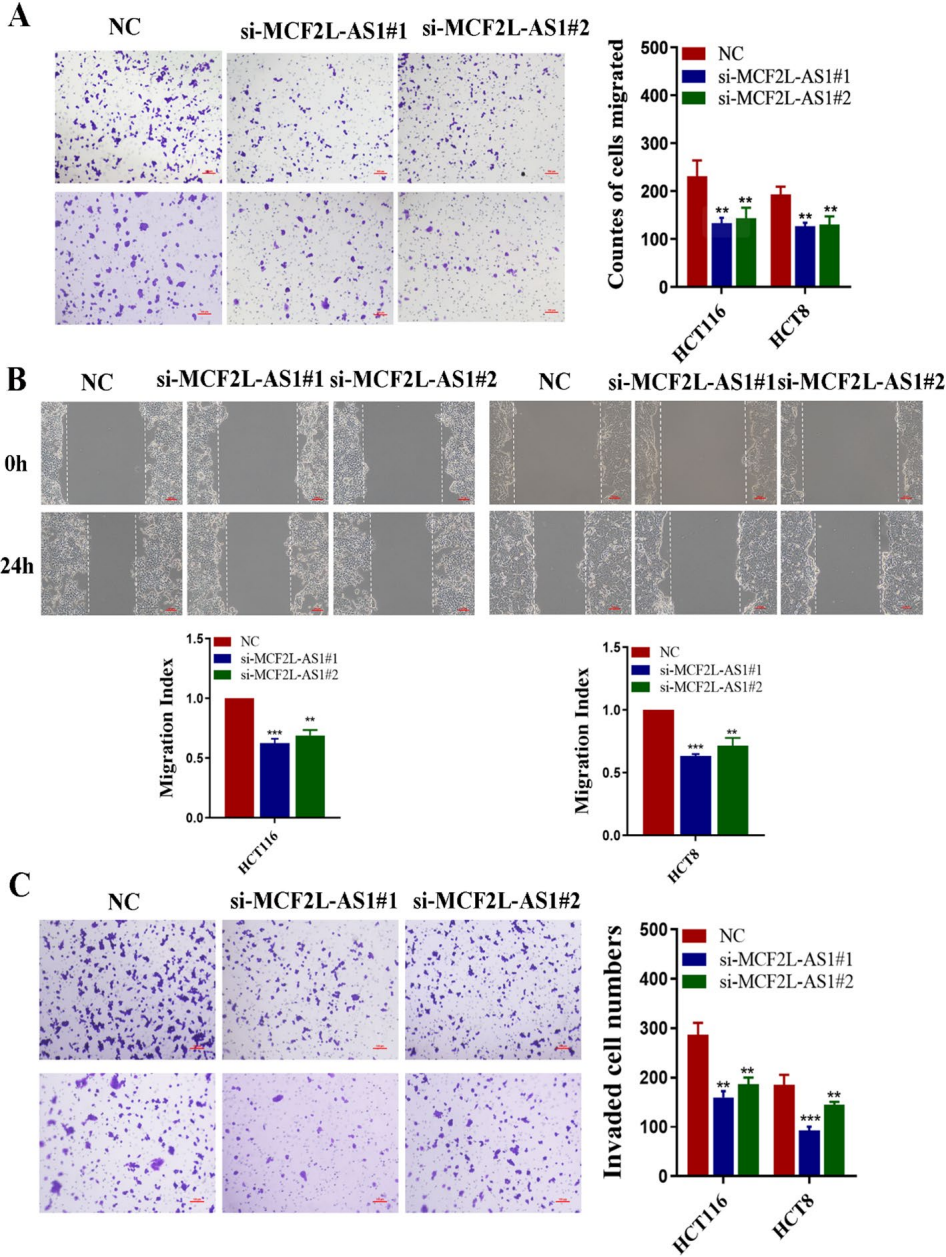
MCF2L-AS1 promotes colorectal cancer cell migration and invasion in vitro. **A** Transwell assays were used to determine the migration abilities of infected cells. **B** Representative images of wound healing assays performed using HCT116 cells and HCT8 cells after MCF2L-AS1 was silenced. **C** Transwell assays were used to determine the invasion abilities of infected cells. Scale bar = 100 μm. *p < 0.05, **p < 0.01, and ***p < 0.001

To comprehensively investigate the functional impact of MCF2L-AS1 on CRC cells migration and invasion, we conducted transwell and wound healing assays to assess the migration potential of HCT116 and HCT8 cells following MCF2L-AS1 suppression. Our results from the transwell assays revealed that down-regulation of MCF2L-AS1 impaired the migration capacity of CRC cells (Fig. 3A). Additionally, findings from the wound healing assay corroborated this conclusion, aligning with those observed in the transwell assay (Fig. 3B). For invasion analysis, matrigel-coated transwell membranes were utilized to evaluate extracellular invasion capability. The data demonstrated that MCF2L-AS1 depletion markedly attenuated the invasive potential of both HCT116 and HCT 8 cells (Fig. 3C). These consistent findings showed that MCF2L-AS1 plays a crucial role in promoting CRC cell migration and invasion in vitro.

### MCF2L-AS1 promotes colorectal cancer cell growth and metastasis in vivo

**Fig. 4:**
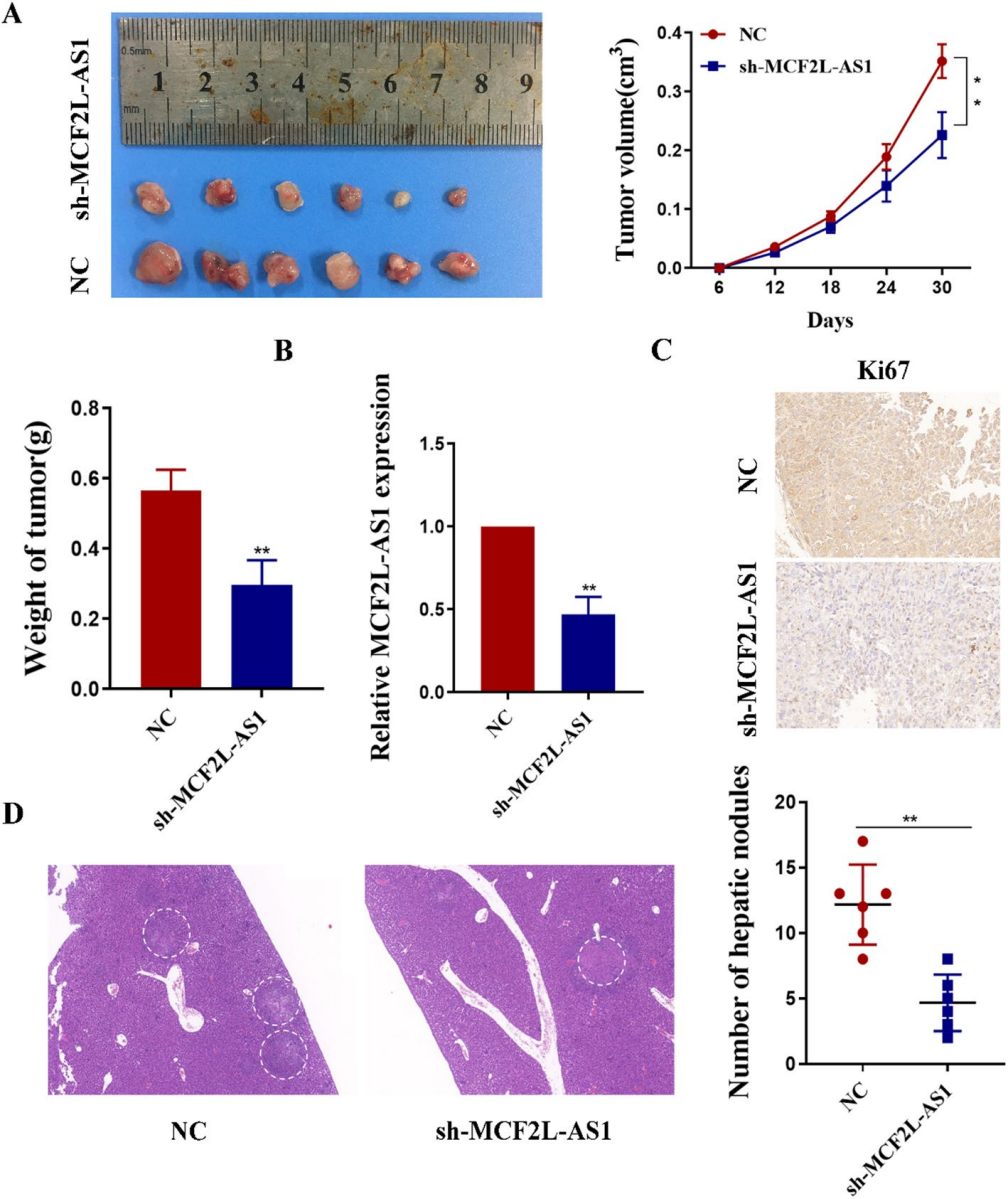
MCF2L-AS1 promotes colorectal cancer cell growth and metastasis in vivo. **A** Representative image of tumors formed in nude mice from empty vector and sh-MCF2L-AS1 vector groups and the tumor volume growth curves and weight of different groups. **B** MCF2L-AS1 expression was detected in tumors from different groups of mice using qRT-PCR. **C** Representative images for H&E-staining, Ki67 immunostaining of tumor samples from the different groups. **D** The number of liver metastatic nodules in nude mice in the three groups. *p < 0.05, **p < 0.01, and ***p < 0.001

To explore whether MCF2L-AS1 promotes the growth and metastasis of CRC cells in vivo as well as in vitro, we conducted a subcutaneous tumor xenograft model as shown in the method. Quantitative analysis of the transplanted tumor development revealed substantial reductions in both weights and volume in the group receiving shRNA-mediated MCF2L-AS1 knockdown compared to the control group (Fig. 4A). Then we performed qRT-PCR validation, and the data confirmed that the expression of MCF2L-AS1 was markedly down-regulated in sh-MCF2L-AS1 group compared with the control group (Fig. 4B). Further immunohistochemical staining was conducted, comparing with MCF2L-AS1 knockdown, Ki67 expression levels were significantly elevated in the control group, which is a recognized marker of proliferation (Fig. 4C). For the liver metastasis assays, compared with the sh-MCF2L-AS1 group, the control group had more liver metastatic nodules on the liver surface. (Fig. 4D).

### MCF2L is a functional target of MCF2L‑AS1 in colorectal cancer

**Fig. 5:**
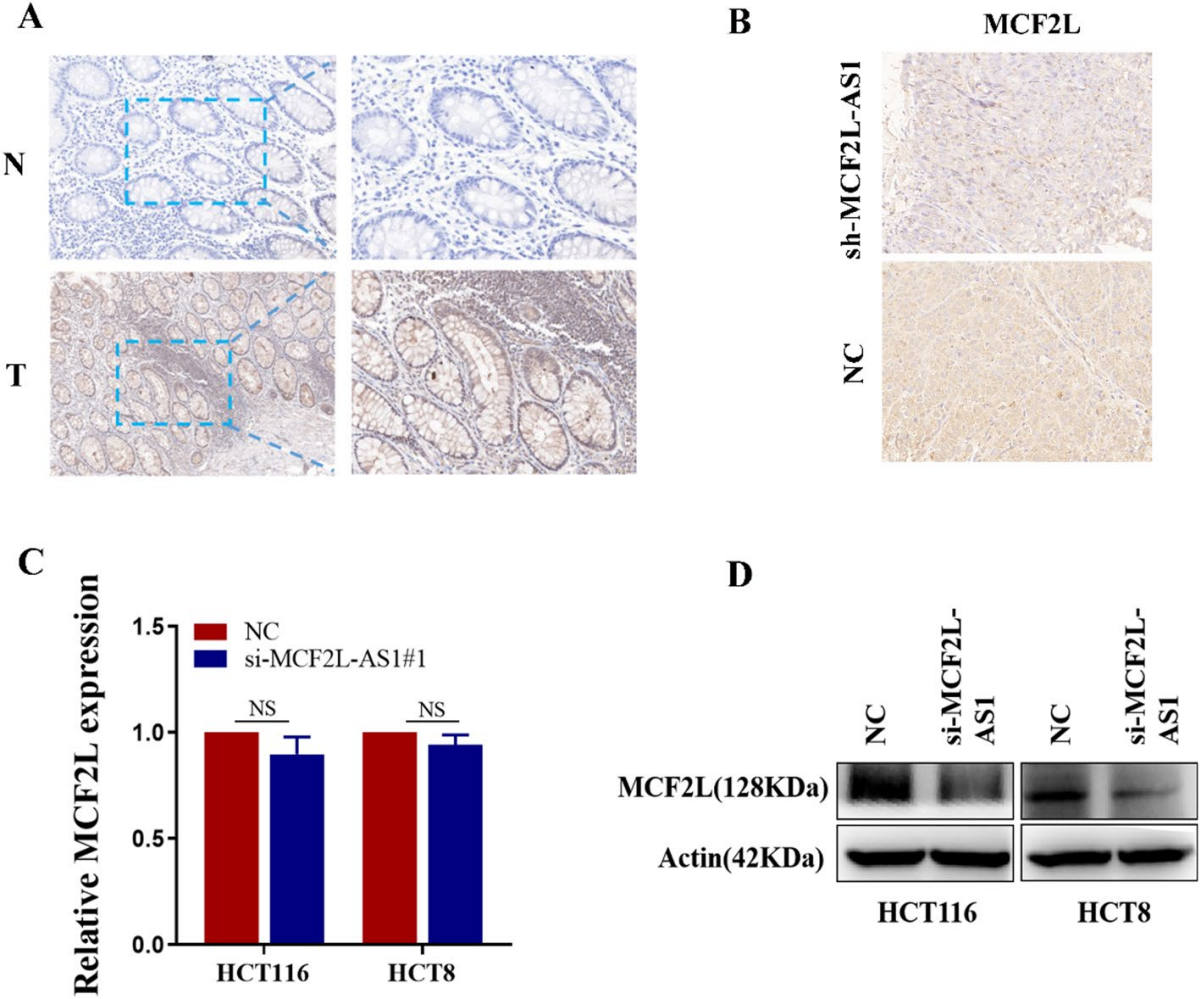
MCF2L is a functional target of MCF2L‑AS1 in colorectal cancer. **A** MCF2L protein levels in CRC tissues and corresponding normal tissues was detected by IHC. **B** MCF2L expression was detected by immunohistochemistry (IHC) xenograft tumor tissues. **C** Quantitative RT-PCR (qRT-PCR) detected the expression of MCF2L in HCT116 and HCT8 cell lines with MCF2L-AS knockdown. **D** Western blot assays detected the expression of MCF2L in HCT116 and HCT8 cell lines with MCF2L-AS knockdown

The biological function of lncRNA is closely related to its intracellular localization. According to our previous experimental results, MCF2L-AS1 is mainly located in the cytoplasm, and previous studies have established that cytoplasmic lncRNAs frequently participate in post-transcriptional regulatory mechanisms [13, 16]. Therefore, we hypothesized that antisense transcript MCF2L-AS1 might modulate MCF2L expression, potentially contributing to colorectal carcinogenesis. Initial immunohistochemical examination of clinical specimens showed elevated MCF2L protein levels in CRC tissues compared to matched normal adjacent tissues (Fig. 5A). IHC assays were also conducted in subcutaneous transplanted tumor tissues, and the results displayed elevated MCF2L protein levels in the control group compared to the sh-MCF2L-AS1 group. (Fig. 5B). Western blot analysis showed that MCF2L was down-regulated in CRC cells with MCF2L-AS1 knockdown. However, qRT-PCR results showed that the down-regulation of MCF2L-AS1 expression level did not affect the mRNA level of MCF2L (Fig. 5C-D).

### MCF2L‑AS1 activates the translation of MCF2L mRNA via recruiting AUF1

AUF1, also known as heteronuclear ribonucleoprotein D (hnRNPD), is a member of the heteronuclear RNA family. Members of the HnRNA family play significant roles in transcriptional regulation, mRNA splicing, output and degradation. By consulting the literature, we learned that AUF1 is involved in various pathological processes and plays a role in apoptosis, immunity, stress, inflammation, aging and tumors. More importantly, AUF1 can bind to the (A + U) rich element in the 3 ‘-untranslated region (3’ -UTR) of the target gene, promoting the translational expression of the target gene without affecting the expression level of the target gene mRNA.

**Fig. 6:**
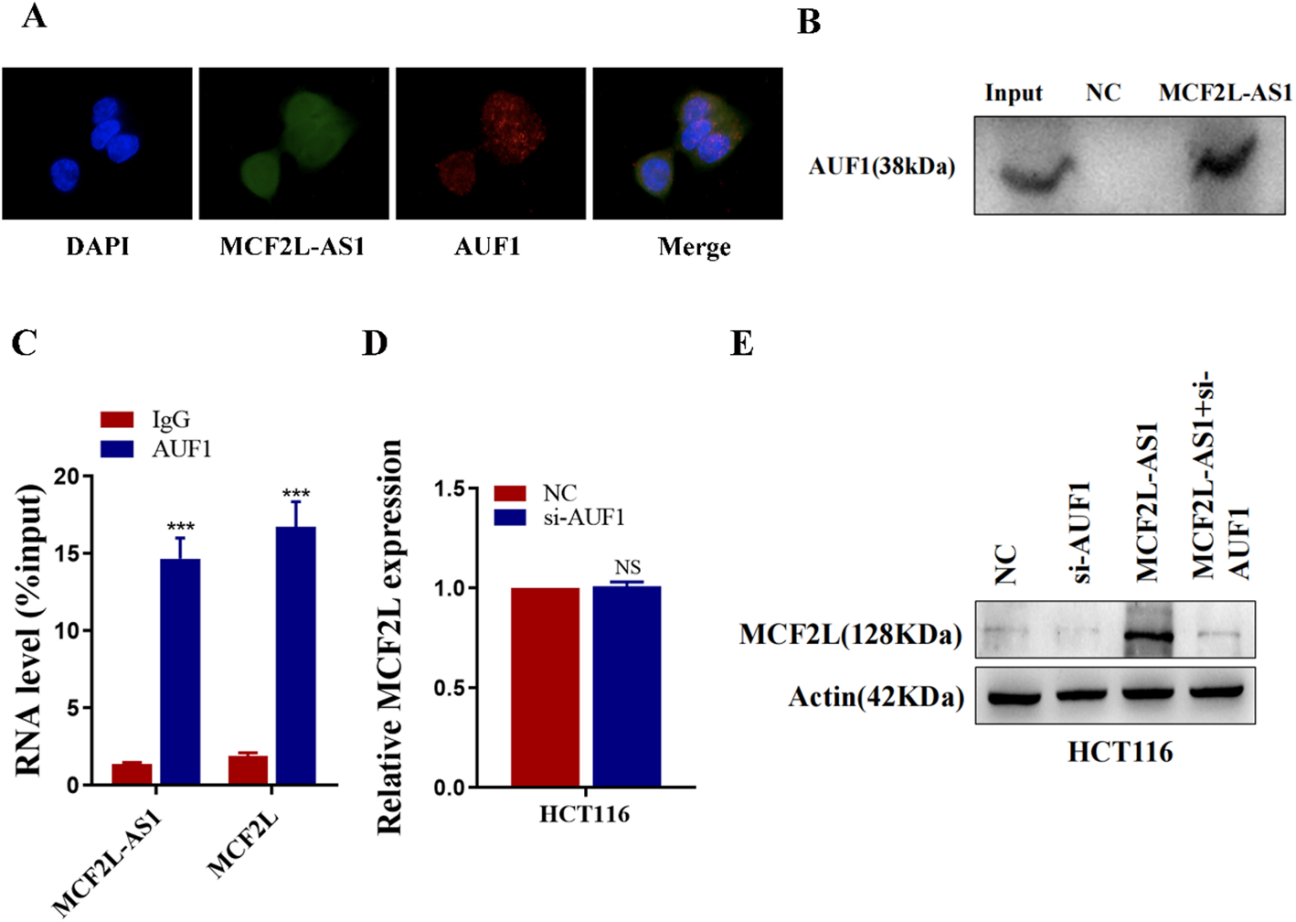
MCF2L‑AS1 activates the translation of MCF2L mRNA via recruiting AUF1. **A** The co-localization of AUF1 and MCF2L-AS1 was assessed by FISH-IF. **B** RNA pull-down assays were used to examine the interaction of MCF2L-AS1 and AUF1 in HCT-116 cells. **C** RNA immunoprecipitation with an anti-AUF1 antibody was used to assess endogenous AUF1binding to RNA in HCT-116 cells; IgG was used as the control. MCF2L-AS1 and MCF2L levels were determined by quantitative RT-PCR (qRT-PCR) and presented as fold enrichment in AUF1 relative to input. **D** qRT-PCR assays detected the expression of MCF2L in HCT116 cells with AUF1 knockdown. **E** MCF2L expression was detected by western blotting in HCT116 cells with indicated treatment. *p < 0.05, **p < 0.01, and ***p < 0.001

 [17–20]. FISH/IF analysis revealed that AUF1 co-located with MCF2L-AS1 in the cytoplasm (Fig. 6A). We used a biotin-labeled MCF2L-AS1 probes for RNA pull-down assays to identify the protein that binds to it. The results confirmed that MCF2L-AS1 could bind to the AUF1 protein in HCT116 cell extracts (Fig. 6B). Meanwhile, we conducted RIP assays to further confirm that MCF2L-AS1 and MCF2L can directly bind to AUF1(Fig. 6C). As depicted in Figs. 6D–E, knockdown of AUF1 leads to a reduction in the protein level of MCF2L without altering its mRNA expression. Furthermore, the reduction in AUF1 partially reverses the altered MCF2L expression induced by MCF2L-AS1 overexpression. These findings suggested that MCF2L-AS1 facilitated translation by binding to AUF1, without altering the mRNA expression of MCF2L.

### Tumor-promoting functions of MCF2L-AS1 are dependent on MCF2L

**Fig. 7:**
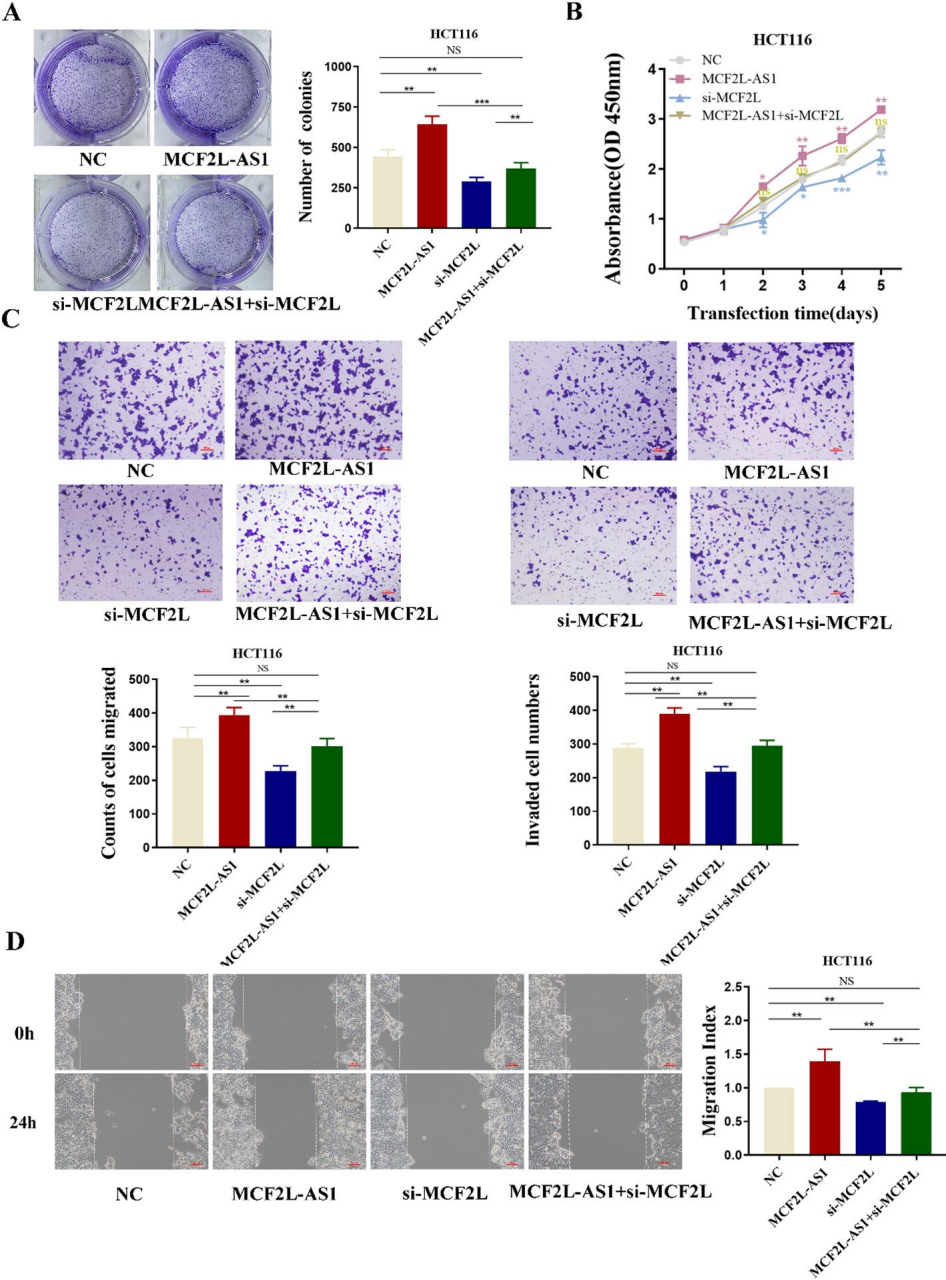
Tumor-promoting functions of MCF2L-AS1 are dependent on MCF2L. **A** Colony formation assays demonstrated that overexpression of MCF2L-AS1 promoted cancer cell growth. MCF2L knockdown could abolish growth promotion caused by MCF2L-AS1. **B** CCK8 assays showed that MCF2L knockdown abolished the increased proliferation rates of colorectal cancer (CRC) cells caused by MCF2L-AS1. **C**, **D** Transwell assays and wound healing assays demonstrated that MCF2L knockdown abolished the increased abilities of migration and invasion caused by MCF2L-AS1. Scale bar = 100 μm. *p < 0.05, **p < 0.01, and ***p < 0.001

To clarify whether the tumor-promoting effect of MCF2L-AS1 in CRC is mediated by MCF2L, we performed several rescue experiments by simultaneously transfecting MCF2L-siRNA into cells overexpressed with MCF2L-AS1. The colony formation assays and CCK8 assay results showed that overexpression of MCF2L-AS1 enhances the proliferation abilities of HCT116 cells, but this effect is reversed by knocking down MCF2L (Fig. 7A–B). As indicated in Fig. 7C–D, in MCF2L-AS1-overexpressing cells, the migration and invasion of cells were significantly up-regulated compared with the control group, but the co-expression of si-MCF2L significantly reversed this effect. In summary, the above results confirm that MCF2L-AS1 exerts its tumor-promoting effect by regulating the expression of MCF2L.

## Discussion

Although the increasingly advanced diagnostic and treatment methods in recent years have to some extent extended the survival of CRC patients, the treatment of CRC remains challenging, due to various factors such as drug resistance, advanced diagnosis, and tumor metastasis. Thus, there is a pressing need for novel molecular biomarkers and therapeutic targets. Our investigation observed a high expression of MCF2L-AS1 in CRC tissues and cell lines. We conducted a series of functional experiments and the data confirmed that MCF2L-AS1 significantly promoted the growth and metastasis of CRC cells *in vivo and in vitro*. Mechanistic studies revealed that cytoplasmic-localized MCF2L-AS1 facilitates AUF1-mediated translational activation of MCF2L mRNA, leading to increased protein synthesis without altering transcriptional levels.

LncRNA is an important molecular marker for human diseases and has been extensively studied. Abnormal lncRNA expression in tumors has been confirmed to impact cellular biological behavior. The abnormal expression of MCF2L-AS1 in tumors has been reported, and it has been identified as a tumor-promoting factor across multiple malignancies, including breast cancer [21], ovarian cancer [22], and hepatocellular carcinoma [23]. Kong et al. reported that MCF2L-AS1 can act as a molecular sponge of miR-105-5p in CRC to regulate the expression of RAB22A and thus accelerate the progression of EMT [24]. Consistent with the data from Kong et al., our findings revealed significant overexpression of MCF2L-AS1 in CRC cells and tissues, and functional validation demonstrated that MCF2L-AS1 can promote the growth and migration of CRC cells both *in vitro and in vivo*. These collective results support the hypothesis that MCF2L-AS1 exerts oncogenic functions in CRC.

Studies have confirmed the close relationship between the subcellular localization of lncRNA and its biological mechanism. In this study, FISH and subcellular fractionation assay showed that lncRNA MCF2L-AS1 was mainly located in the cytoplasm of CRC cells, suggesting that the mechanism of action promoting the development of CRC may be regulated at the post-transcriptional levels. MCF2L‑AS1 is a noncoding antisense transcript produced by MCF2L promoters, located at the physical adjacency site of MCF2L. Notably, this study confirmed that MCF2L-AS1 positively regulates MCF2L at protein levels, but not at mRNA levels. To elucidate the regulatory mechanism through which MCF2L-AS1 modulates MCF2L protein abundance, the protein of MCF2L-AS1 interaction was investigated. AUF1 is an RNA-binding protein that generates four transcript variants after alternative pre-messenger RNA (pre-mRNA) splicing. It has a typical role in controlling the stability or translation of mRNA targets, based on the recognition of AU-rich sequences in the 3’-UTE of target mRNA. The result of FISH/IF showed that AUF1 co-located with MCF2L-AS1 in the cytoplasm, and the direct binding of AUF1 and MCF2L-AS1 was confirmed by RIP and RNA pull- down assay. Further, we confirmed that the down-regulation of AUF1 expression did not affect the mRNA level of MCF2L, but led to the down-regulation of its protein level, and could reverse the up-regulation of MCF2L expression caused by the overexpression of MCF2L-AS1. In addition, we confirmed through a series of functional experiments that the tumor-promoting effect of MCF2L-AS1 in CRC is through regulating the expression of MCF2L.

Overall, these findings collectively demonstrated that MCF2L-AS1 exerts critical oncogenic functions during colorectal carcinogenesis through AUF1-mediated translational activation of MCF2L mRNA. Enhancing the understanding of MCF2L-AS1’s role in CRC progression and its mechanism not only contributes to a better comprehension of lncRNA-mediated tumor development, but also facilitates the identification of novel molecular markers for tumors and the development of therapeutic strategies.

## Supplementary Information

Supplementary Material 1.

## Data Availability

The data used to support the findings of this study are available from the corresponding author upon request.

## References

[1] Siegel RL, Miller KD, Fuchs HE, Jemal A. Cancer statistics, 2022. CA Cancer J Clin. 2022;72(1):7–33. 35020204 10.3322/caac.21708

[2] Deniz E, Erman B. Long noncoding RNA (lincRNA), a new paradigm in gene expression control. Funct Integr Genomics. 2017;17(2–3):135–43. 27681237 10.1007/s10142-016-0524-x

[3] Loewen G, Jayawickramarajah J, Zhuo Y, Shan B. Functions of lncRNA HOTAIR in lung cancer. J Hematol Oncol. 2014;7:90. 25491133 10.1186/s13045-014-0090-4 PMC4266198

[4] Kong J, Sun W, Li C, Wan L, Wang S, Wu Y, et al. Long non-coding RNA LINC01133 inhibits epithelial-mesenchymal transition and metastasis in colorectal cancer by interacting with SRSF6. Cancer Lett. 2016;380(2):476–84. 27443606 10.1016/j.canlet.2016.07.015

[5] Lee JT. Epigenetic regulation by long noncoding RNAs. Science. 2012;338(6113):1435–9. 23239728 10.1126/science.1231776

[6] Fatica A, Bozzoni I. Long non-coding RNAs: new players in cell differentiation and development. Nat Rev Genet. 2014;15(1):7–21. 24296535 10.1038/nrg3606

[7] Nakagawa S, Kageyama Y. Nuclear lncRNAs as epigenetic regulators-beyond skepticism. Biochim Biophys Acta. 2014;1839(3):215–22. 24200874 10.1016/j.bbagrm.2013.10.009

[8] Kornienko AE, Guenzl PM, Barlow DP, Pauler FM. Gene regulation by the act of long non-coding RNA transcription. BMC Biol. 2013;11:59. 23721193 10.1186/1741-7007-11-59 PMC3668284

[9] Ni W, Zhang Y, Zhan Z, Ye F, Liang Y, Huang J, et al. A novel lncRNA uc.134 represses hepatocellular carcinoma progression by inhibiting CUL4A-mediated ubiquitination of LATS1. J Hematol Oncol. 2017;10(1):91. 28420424 10.1186/s13045-017-0449-4 PMC5395742

[10] Zhao Y, Du T, Du L, Li P, Li J, Duan W, et al. Long noncoding RNA LINC02418 regulates MELK expression by acting as a ceRNA and may serve as a diagnostic marker for colorectal cancer. Cell Death Dis. 2019;10(8):568. 31358735 10.1038/s41419-019-1804-x PMC6662768

[11] Zhu C, Cheng D, Qiu X, Zhuang M, Liu Z. Long noncoding RNA SNHG16 promotes cell proliferation by sponging microRNA-205 and upregulating ZEB1 expression in osteosarcoma. Cell Physiol Biochem. 2018;51(1):429–40. 30453308 10.1159/000495239

[12] Xu M, Chen X, Lin K, Zeng K, Liu X, Pan B, et al. The long noncoding RNA SNHG1 regulates colorectal cancer cell growth through interactions with EZH2 and miR-154-5p. Mol Cancer. 2018;17(1):141. 30266084 10.1186/s12943-018-0894-x PMC6162892

[13] Chen DL, Lu YX, Zhang JX, Wei XL, Wang F, Zeng ZL, et al. Long non-coding RNA UICLM promotes colorectal cancer liver metastasis by acting as a ceRNA for microRNA-215 to regulate ZEB2 expression. Theranostics. 2017;7(19):4836–49. 29187907 10.7150/thno.20942 PMC5706103

[14] Zhang Z, Yang W, Li N, Chen X, Ma F, Yang J, et al. LncRNA MCF2L-AS1 aggravates proliferation, invasion and glycolysis of colorectal cancer cells via the crosstalk with miR-874-3p/FOXM1 signaling axis. Carcinogenesis. 2021;42(2):263–71. 32860508 10.1093/carcin/bgaa093

[15] Cai M, Hu W, Huang C, Zhou C, Li J, Chen Y, et al. lncRNA MCF2L-AS1/miR-105/ IL-1β Axis Regulates Colorectal Cancer Cell Oxaliplatin Resistance. Cancer Manag Res. 2021;13:8685–94. 34824551 10.2147/CMAR.S313905 PMC8610381

[16] Lu QC, Rui ZH, Guo ZL, Xie W, Shan S, Ren T. LncRNA-DANCR contributes to lung adenocarcinoma progression by sponging miR-496 to modulate mTOR expression. J Cell Mol Med. 2018;22(3):1527–37. 29266795 10.1111/jcmm.13420 PMC5824415

[17] Moore AE, Chenette DM, Larkin LC, Schneider RJ. Physiological networks and disease functions of RNA-binding protein AUF1. WIREs RNA. 2014;5(4):549–64. 24687816 10.1002/wrna.1230

[18] Yoon JH, De S, Srikantan S, Abdelmohsen K, Grammatikakis I, Kim J, et al. PAR-CLIP analysis uncovers AUF1 impact on target RNA fate and genome integrity. Nat Commun. 2014;5:5248. 25366541 10.1038/ncomms6248 PMC4291169

[19] White EJ, Matsangos AE, Wilson GM. AUF1 regulation of coding and noncoding RNA. Wiley Interdiscip Rev RNA. 2017. 10.1002/wrna.1393. 27620010 10.1002/wrna.1393 PMC5315606

[20] Kumar M, Matta A, Masui O, Srivastava G, Kaur J, Thakar A, et al. Nuclear heterogeneous nuclear ribonucleoprotein D is associated with poor prognosis and interactome analysis reveals its novel binding partners in oral cancer. J Transl Med. 2015;13:285. 26318153 10.1186/s12967-015-0637-3 PMC4553214

[21] She Q, Chen Y, Liu H, Tan J, Li Y. A high level of the long non-coding RNA MCF2L-AS1 is associated with poor prognosis in breast cancer and MCF2L-AS1 activates YAP transcriptional activity to enhance breast cancer proliferation and metastasis. Bioengineered. 2022;13(5):13437–51. 36700469 10.1080/21655979.2022.2074108 PMC9276029

[22] Zhu Y, Yang L, Wang J, Li Y, Chen Y. SP1-induced lncRNA MCF2L-AS1 promotes cisplatin resistance in ovarian cancer by regulating IGF2BP1/IGF2/MEK/ERK axis. J Gynecol Oncol. 2022;33(6):e75. 36245227 10.3802/jgo.2022.33.e75 PMC9634094

[23] Ou H, Qian Y, Ma L. MCF2L-AS1 promotes the biological behaviors of hepatocellular carcinoma cells by regulating the miR-33a-5p/FGF2 axis. Aging Albany NY. 2023;15(13):6100–16. 37432067 10.18632/aging.204795 PMC10373981

[24] Kong W, Li H, Xie L, Cui G, Gu W, Zhang H, et al. LncRNA MCF2L-AS1 aggravates the malignant development of colorectal cancer via targeting miR-105-5p/RAB22A axis. BMC Cancer. 2021;21(1):1069. 34592939 10.1186/s12885-021-08668-w PMC8482615

