# Supplementary material for: LncRNA MCF2L-AS1 promotes malignant progression of colorectal cancer by post-transcriptional activation of MCF2L
Source: Discov Oncol. 2026 Jan 8;17:241. doi: 10.1007/s12672-026-04394-6 (PMC12873018; doi:10.1007/s12672-026-04394-6)
Supplement: Supplementary file 1 — Supplementary Material 1. [file 12672_2026_4394_MOESM1_ESM.docx]

**Supplementary Materials and Methods**

**RNA Extraction and Quantitative Real-Time PCR**

According to the manufacturer’s protocol, total RNA was extracted from cells and tissues by using TRIzol reagent (Invitrogen, USA). CircRNA and mRNA detection was conducted using a SYBR Green PCR Kit (Takara, Japan) with an ABI 7500 System. GAPDH was used as an internal control. The expression levels of miRNAs were carried out using Hairpin-it microRNA and U6 snRNA Normalization RT-PCR Quantitation Kit (Genepharma, China). For copy number detection, total RNA was reversely transcribed into complementary DNA (cDNA) using PrimeScriptRT reagent kit (Takara, Japan), and qRT-PCR was performed with TB Green® Premix Ex Taq™ II (Tli RNaseH Plus) (TaKaRa, China) on ABI 7500 real-time PCR system (Applied Biosystems, CA, USA). Fold changes were analyzed using the 2-ΔΔCT method. The primers sequences were as follows:

5’-CTCGCTTCGGCAGCACA-3’ and 5’-AACGCTTCACGAATTTGCGT -3’ for U6; 5’-GGGAGCCAAAAGGGTCATCA -3’ and 5’- TGATGGCATGGACTGTGGTC-3’ for GAPDH; 5’-GATCAACGTTCAATCCACCG -3’ and 5’ ACGTCAAGATAGCGCAGCTTCC-3’ for MCF2L-AS1; 5’-AAACCGAGGCTGCCTTCGATGA -3’ and 5’-TGCCGATGTCTGTGAAGGTTGC3’ for MCF2L;

**In vivo experiments**

All experiments animal were raised in an environment free of specific pathogens in accordance with a protocol approved by the Animal Care Committee of Nanjing Medical College. For the subcutaneous tumor xenograft model, male BALB/c nude mice (6 weeks old) were randomly divided into two groups with six mice in each group. Stably infected HCT116 cells or negative control (5 × 10^6^ cells/0.2 mL PBS) were inoculated into the left and right upper arm of the same nude mice, respectively. Measured the size of the subcutaneous tumor with caliper measurements every 5 days and calculated the tumor volumes according to the following formula: (L × W2)/2. A total of four weeks later, all mice were euthanized via intraperitoneal injection of 3% pentobarbital sodium (Sigma, Germany), their tumors excised, and subsequently weighed.

For the lung tumor metastasis model, male BALB/c nude mice (6 weeks old) were randomly divided into two groups with four mice in each group. HCT116 cells stably infected with sh-MCF2L-AS1 or empty vectors suspended with PBS (1×10^6^cells/0.1ml PBS) were injected into the lateral tail vein of the nude mice using a 1ml sterile syringe. A total of 4 weeks later, all mice were euthanized via intraperitoneal injection of 3% pentobarbital sodium (Sigma, Germany), and the lung tissues were extracted and soaked in 4% paraformaldehyde solution for HE staining.

**RNA immunoprecipitation assay**

The RNA immunoprecipitation assay was conducted using the EZ Magna RNA immunoprecipitation Kit (Millipore) was used according to the manufacturer's protocol. First, magnetic beads were preincubated with anti-AUF1 antibody. HCT-116 cells (1x10^7^) were lysed in RNA immunoprecipitation (RIP) lysis buffer and incubated with magnetic beads at 4°C overnight after one freeze-thaw cycle. The RNA was extreacted, and the relative expression of MCF2L-AS1 and MCF2L was measured by qRT-PCR. IgG was used as the negative control and and normalized to the relative expression of MCF2L-AS1 and MCF2L in input samples.

**RNA pull-down assay**

The RNA pull-down assays were performed using a Magnetic RNA Protein Pull-Down Kit (Thermo Fisher Scientific). CRC cells (2 x10^7^) were cross-linked for each hybridization reaction. Briefly, the biotin-labeled MCF2L-AS1 RNA probes were incubated with cell lysates at 4°C overnight. The binding proteins were isolated by western blot electrophoresis.

**Apoptosis Assay**

Apoptosis assays were performed using the Annexin V-FITC apoptosis detection kit (KeyGEN) following the instructions. Briefly, after transfections, cells were collected and washed with cold PBS at 4 ℃ twice, followed by treatment with Annexin V-FITC and propidium iodide (PI) in the dark for 15 minutes at room temperature. A FACSCalibur flow cytometer (BD Biosciences, USA) was used to detect apoptosis.
